# Undiagnosed Insulinoma in a Young Patient With Idiopathic Dilated Cardiomyopathy: A Case Report

**DOI:** 10.7759/cureus.81674

**Published:** 2025-04-03

**Authors:** Rosaida Silverio Lopez, Justin Owens, Mariya Tom, Saif Borgan, Megan Gallagher, Edward Distler

**Affiliations:** 1 Internal Medicine, South Georgia Medical Center, Valdosta, USA; 2 Endocrinology, South Georgia Medical Center, Valdosta, USA; 3 Cardiology, South Georgia Medical Center, Valdosta, USA

**Keywords:** cardiomyopathy, insulinoma, neuroendocrine tumor, obesity, reduced ejection fraction

## Abstract

Insulinomas are rare pancreatic tumors that present with symptoms of hypoglycemia secondary to unregulated high levels of insulin. Literature has described that recurrent hypoglycemic events induce a sympathetic drive that could compromise cardiac function. Tumor resection eliminates the hypoglycemia source, halting the cycle of sympathetic overdrive and improving cardiac function. We present the case of a 43-year-old patient who was hospitalized after a recent gastric bypass for recurrent episodes of confusion, mumbling, night terrors, and low glucose levels. She also had a chronic diagnosis of idiopathic dilated cardiomyopathy and heart failure with an ejection fraction of 30%. Imaging and laboratory studies demonstrated the presence of insulinoma. She underwent tumor resection with improvement in her exercise tolerance. Her ejection fraction improved from 30% to 35-40% eight months post-resection. This case is intended to review current data on the possible association between insulinomas and heart disease. To the best of our knowledge, our report stands out as one of the few case studies available that explores this potential association. It is crucial to recognize that tumor resection can significantly enhance cardiac function, making it imperative to thoroughly investigate the diverse cardiovascular effects linked to insulinomas.

## Introduction

Insulinomas are rare neuroendocrine tumors arising from the pancreatic islets of Langerhans and can present as both sporadic or hereditary conditions commonly associated with multiple endocrine neoplasia type 1 (MEN1) [1]. These tumors produce and autonomously secrete excessive amounts of insulin. Insulin plays a critical role in metabolic and hemodynamic regulation. Unregulated hypersecretion of insulin into the blood causes resultant episodes of hypoglycemia. Neuroglycopenic symptoms may present, such as vision changes, confusion, seizures, or even coma in severe cases. Hypoglycemia, in turn, exerts hemodynamic effects through the body's adrenergic response. These include increased sympathetic activity, coronary blood flow, and decreased afterload [2,3]. It is thought that the chronic hyperinsulin state causes chronic sympathetic activation affecting both the heart and vasculature, suggesting a potential link between hyperinsulinemic hypoglycemia and cardiovascular morbidity [4]. Chronic activation of the sympathetic nervous system (SNS) leads to excessive stimulation of β1-adrenergic receptors in the heart, which increases intracellular cyclic adenosine monophosphate (AMP) and calcium overload, which leads to myocyte apoptosis, oxidative stress, and mitochondrial dysfunction [5]. Over time, these effects cause myocardial remodeling and ventricular dilation. Sympathetic activation also leads to peripheral vasoconstriction via alpha 1-adrenergic receptors, increasing systemic vascular resistance, which raises afterload, forcing the heart to work harder to pump blood, leading to progressive ventricular dilation and systolic dysfunction. This is further supported by the U-shaped curve that depicts the relationship between hemoglobin A1c (HbA1c) levels and the risk of death. Both low and high HbA1c levels are associated with increased mortality, more so in patients with cardiovascular disease [6]. Insulinomas have been associated with rare complications, including cardiomyopathy. The mechanism behind this is thought to be secondary to the biochemical implications of insulin on cardiac cells and the sympathetic drive caused by repeated hypoglycemia episodes.

## Case presentation

A 43-year-old female was brought to the hospital by Emergency Medical Services (EMS) because of altered mental status. Her partner found the patient confused, mumbling, and having purposeless movements. En route to the hospital, a point of care fingerstick glucose was low at 28 mg/dL, and she received an ampule of 50% dextrose and was started on a 10% dextrose infusion. She described similar but milder episodes happening approximately once a month, usually at night, since her teenage years, which were assumed to be night terrors. The patient did not have a history of diabetes and did not take any oral hypoglycemic agents except for dapagliflozin as part of Guideline-Directed Medical Therapy (GDMT) for heart failure with reduced ejection fraction (HFrEF).

The patient had been recovering from a gastric bypass procedure five days before presentation to address her obesity and was not able to consume her usual high-carbohydrate diet. She was the only member of her family with obesity, and she described a long history of carbohydrate cravings, which had contributed to her weight gain.

Her symptoms were significantly accentuated in frequency and severity by her gastric bypass surgery. She reported chronic heart failure symptoms, including orthopnea, reduced exercise tolerance, and constant fatigue. Her physical exam showed a normal heart rate with a regular rhythm and no cardiac murmurs or rubs. She had no pulmonary rales or wheezing. No focal neurological deficits were noticed on examination. Her abdomen showed healing laparoscopic incisions that appeared well-approximated with no bleeding or infection. She was not in acute heart failure exacerbation at the time of hospitalization.

Past medical history

This patient was previously diagnosed with idiopathic HFrEF (initially 30% and subsequently 34%) and non-ischemic cardiomyopathy. Her records described the presence of HFrEF since 2021, two years before her hospitalization. She also had obesity, hypertension, obstructive sleep apnea with the use of a continuous positive airway pressure device, anxiety, hyperlipidemia, and asthma. The patient had no known history of diabetes mellitus or prediabetes (HbA1c was 5% one year before hospitalization). She was receiving GDMT with sacubitril/valsartan, metoprolol, dapagliflozin, and Aldactone. Metoprolol is a β1-selective adrenergic blocker that can reduce the autonomic warning signs of hypoglycemia by blocking receptors in the heart, suppressing symptoms such as tachycardia, tremors, and palpitations. Metoprolol and dapagliflozin were temporarily held during her hospitalization to avoid masking or worsening hypoglycemic symptoms.

Differential diagnosis

During our clinical evaluation, multiple possible diagnoses were considered, including noninsulinoma pancreatogenous hypoglycemia syndrome (NIPHS), insulinoma, insulin autoimmune syndrome (IAS), post-gastric bypass hypoglycemia (PGBH), undisclosed use of exogenous insulin or oral hypoglycemic agents, and hypoglycemia due to inadequate oral intake. Considering the patient's laboratory results described in Table 1, our clinical suspicion for these various entities was more inclined toward insulinoma. The high levels of proinsulin, insulin, and c-peptide strongly suggested the diagnosis of insulinoma and made other diagnoses like NIPH or PGBH still probable. Low beta-hydroxybutyrate also suggested suppressed fat metabolism due to excessive insulin activity, such as insulinoma. A negative sulfonylurea screen and negative insulin antibody helped rule out oral hypoglycemic agents-induced hypoglycemia and IAS. Exogenous use of insulin was improbable, considering the elevated C-peptide.

**Table 1:** Insulin studies after fasting period without dextrose. Elevated insulin, pro-insulin, and C-peptide levels, along with low β-hydroxybutyrate, suggest a possible insulinoma. TSH: thyroid-stimulating hormone

Test	Result	Reference Range
Glucose	41 mg/dL	70-100 mg/dL
Insulin	38 mg/dL	2.6-24.9 mg/dL
C-peptide	7.0 ng/mL	1.1-4.4 ng/mL
Proinsulin	112 pmol/L	3.6-22 pmol/L
B-hydroxybutyrate	<0.10 mmol/L	≤0.40 mmol/L
Cortisol	7 µg/dL	5-23 µg/dL
TSH	0.87 mU/L	0.45-4.5 mU/L
Sulfonylurea screen	Negative	Negative
Insulin antibody	Negative	Negative

Investigations

**Figure 1:**
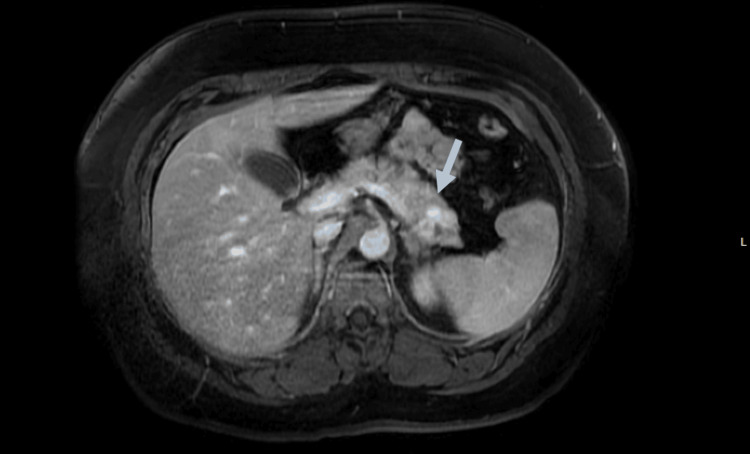
Trilobed pancreatic mass indicated by the white arrow on the abdominal magnetic resonance imaging (MRI).

A hypoglycemia workup was performed after discontinuation of dextrose in a fasting state (see Table 1). A computerized tomography (CT) of the abdomen with contrast was normal. A magnetic resonance imaging (MRI) of the abdomen showed a trilobed pancreatic mass in the pancreatic tail (see Figure 1), consistent with a possible pancreatic islet tumor, likely insulinoma. Pathology confirmed this.

**Figure 2:**
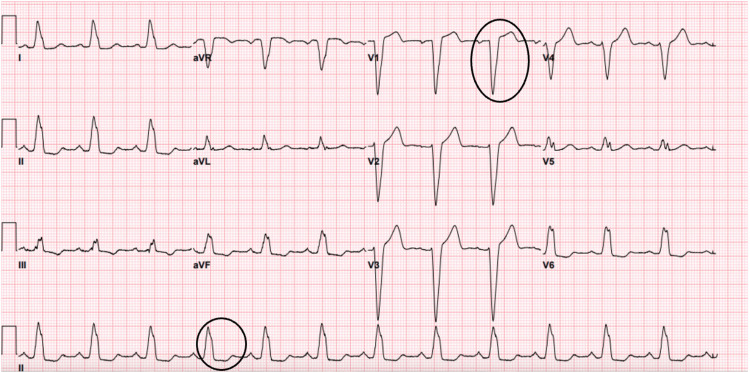
Electrocardiogram on hospital admission showed sinus rhythm and left bundle branch block, which was also present in previous electrocardiogram studies, and prolonged corrected QT interval at 485 milliseconds.

**Figure 3:**
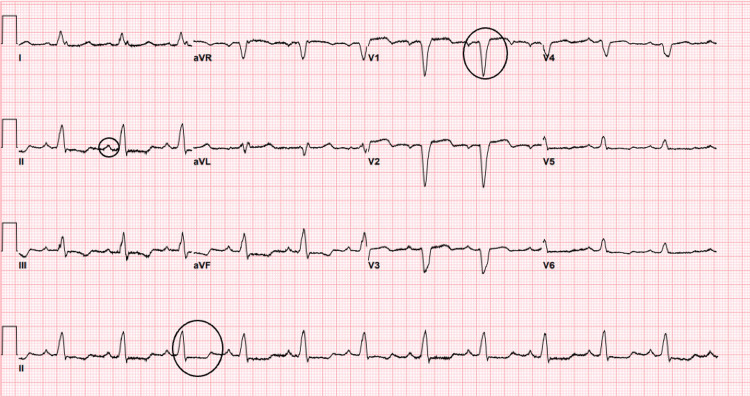
Electrocardiography two months prior to hospital admission showed sinus rhythm, chronic left bundle branch block, prolonged corrected QT interval at 555 milliseconds, and possible left atrial enlargement.

Electrocardiography on admission showed sinus rhythm and left bundle branch block (LBBB) (see Figure 2). A previous electrocardiogram showed the presence of an LBBB. Possible left atrial enlargement was also noticed (see Figure 3).

Excess insulin promotes myocardial fibrosis by activating the insulin-like growth factor-1 (IGF-1). Fibrosis in conduction pathways (especially the left bundle branch) can contribute to LBBB. Chronic insulin excess forces glucose into peripheral tissues, especially muscle and adipose tissue, reducing lipolysis and promoting fat storage, creating an anabolic state that favors adipogenesis. Higher visceral fat promotes chronic inflammation via cytokines like tumor necrosis factor-α (TNF-α) and interleukin 6 (IL-6), impairing glucose regulation. Obesity blunts counterregulatory hormone responses (glucagon, epinephrine, cortisol), increasing the risk of hypoglycemia unawareness and neuroglycopenia [7].

Management

Pending the results of the initial laboratory workup, the patient was started on therapy to reduce her reliance on the 10% dextrose drip, which was administered at a rate of 100 mL/hour. After 48 hours of octreotide (50 mcg three times a day), there was no considerable change in her dextrose drip requirements. Hence, she was switched to diazoxide and acarbose, which allowed us to discontinue the dextrose drip and reduce the frequency of glucose fingerstick checking. Diazoxide stimulates hepatic gluconeogenesis and glycogenolysis, helping to maintain blood glucose and controlling hypoglycemic episodes by reducing excessive insulin secretion, although it can cause fluid retention and hirsutism. Acarbose, an α-glucosidase inhibitor, delays carbohydrate breakdown and absorption in the intestine, which helps prevent reactive hypoglycemia.

After evaluation of her imaging findings, she underwent a spleen-preserving distal pancreatectomy, and a pathology report confirmed her tumor was neuroendocrine, specifically insulinoma. Diazoxide was discontinued, and glucose levels improved postoperatively without additional therapy, with values between 115 and 202 mg/dL and unmasking prediabetes. Postoperative prediabetes in insulinoma patients is due to chronic beta-cell adaptation, insulin resistance, and counterregulatory dysfunction. It is often transient and may improve over weeks to months as the body readjusts its insulin-glucose balance [8].

Follow-up

The patient was seen for a follow-up by endocrinology. Her glucose levels had stabilized. Her inpatient team contacted her five months after hospital discharge. She reported improvement in heart failure symptoms, including the resolution of fatigue and orthopnea. She also had increased exercise tolerance. During a follow-up with the cardiology department six months after the initial hospital visit, the patient reported that she had been compliant with GDMT therapy and had remained asymptomatic for cardiovascular symptoms. The progression of her echocardiography ejection fraction (EF) was inconsistent. In her initial echocardiogram dated 03/27/2023, her EF was 30%. On 07/20/2023, an echocardiogram showed her EF had slightly improved to 35-40%. Five days after the resection of her insulinoma, her EF was 34%. Eight months after tumor resection, the EF was measured at 35-40%. Although there was no significant improvement in EF following her insulinoma resection, further echocardiography studies are required to continue evaluating her response. The current literature does not specify an expected timeframe for EF recovery in patients with insulinoma resection. Still, compared to the recommended guidelines by the American College of Cardiology in patients with heart failure with recovered ejection fraction (HFrecEF), these patients should perhaps be followed in six months to one year. Their EF should be reassessed every three to five years to monitor left ventricular function [9].

## Discussion

A key feature for diagnosing hypoglycemic disorders such as insulinomas is Whipple's triad [10]. Whipple's triad is defined as symptoms of hypoglycemia at the time of measurable low plasma glucose (<70 mg/dL), which resolves after correction of low blood glucose. It is important to note that hypoglycemia alone is not diagnostic of insulinoma, as other disorders and/or malignancies can cause hypoglycemia [11]. The typical presenting characteristic of an insulinoma is fasting hypoglycemia, which results from excessive endogenous insulin release. Fasting hypoglycemia, however, is not all-inclusive, as literature also suggests that endogenous hyper-insulinemic hypoglycemia can present in postprandial states of patients with functioning insulinomas [10]. Other causes of postprandial hypoglycemic states include dumping syndrome in the setting of post-gastric bypass [12]. This was less likely in our patient, who had a gastric sleeve that did not bypass the pyloric sphincter or the duodenum. The pyloric sphincter remains intact in the gastric sleeve, allowing regulated gastric emptying. In contrast, gastric bypass completely bypasses the pylorus, causing uncontrolled rapid emptying of hyperosmolar food into the small intestine, triggering dumping syndrome. Additionally, our patient had more notable fasting than postprandial hypoglycemia, which argues against a reactive response.

The diagnosis of insulinoma is often delayed because of non-specific symptoms, poor provider awareness of this condition, and complex testing algorithms. The annual incidence of insulinoma is about 1-4 cases per million people per year, with a prevalence of 1 in 250,000 people [13]. A hypoglycemia workup must be performed when plasma glucose levels are low. This presents a challenge due to limited resources. Most providers don't feel comfortable admitting patients for a 72-hour fast test and fail to refer patients to endocrinology early in the course of the disease. Imaging studies also have low sensitivity, and often, patients will need multiple localizing studies, such as CT or MRI, or occasionally invasive diagnostic studies, such as endoscopic ultrasound and calcium scan. The sensitivity of multiphasic contrast-enhanced CT for detecting insulinomas ranges from 50% to 80%. Poorly visible small tumors (less than 1 cm) may decrease sensitivity. The sensitivity of MRI is around 85% (more significant for small tumors). Endoscopic ultrasound is excellent for tiny, deep-seated insulinomas, with a sensitivity of >85% to 95% [14].

The gold standard diagnostic test for insulinoma has been the supervised 72-hour fast. However, recent studies show that 90-95% of patients with insulinoma will have a positive test in the first 48 hours, which is becoming the new standard [13]. Surgical treatment of insulinoma starts with proper localization of the lesion. Surgical resection has high cure rates. In patients without localizing lesions or in poor surgical candidates, medical treatment options, such as diazoxide, octreotide, and acarbose, are available to directly or indirectly reduce insulin secretion.

The survival rate of insulinomas depends on the type of insulinoma described. If non-malignant and/or not associated with MEN1, a 10-year survival rate of insulinomas if surgically resected has been reported at 88%, and 87.5% of overall patients are considered cured. This percentage is significantly reduced for malignant insulinomas; the overall survival rate is 24% [14].

In Scherrer and Sartori [3], studies on insulin's cardiovascular influence describe the association of hyperinsulinemia and ischemic heart disease. Systemic insulin infusion in humans causes norepinephrine overflow in the capillaries of the skeletal muscle. Insulin-stimulated nitric oxide synthesis and glucose transport in endothelial cells may use similar signaling mechanisms. It is plausible that a genetically derived or acquired abnormality in this shared signaling system could serve as a scientific foundation for insulin resistance in both vascular and metabolic contexts. Endothelial dysfunction has been linked to insulin-resistant states, and the ensuing endothelial dysfunction may cause the insulin's pressor and depressor activities to be more pronounced, which would ultimately lead to hypertension and increased cardiovascular morbidity.

In a case report presented by Thirumalai et al. [15], a patient with cardiac arrest was found to have hypoglycemia. A cardiac catheterization showed an EF of 26% with angiographically normal coronary arteries. She was found to have an insulinoma, which was described as a possible cause of cardiomyopathy in her presentation. Her EF improved to 41% four months after pancreaticoduodenectomy. The authors hypothesized that dilated cardiomyopathy in the setting of insulinoma could be secondary to the effect of high insulin levels on cardiac ATP-sensitive potassium (KATP) channels. They also described possible ventricular remodeling secondary to the catecholamine surge that happens in hypoglycemia. With the presence of hypoglycemia, patients could present QT segment prolongation, which may precipitate recurrent arrhythmias.

Bienengraeber et al. [16] described a molecular correlation to the development of dilated cardiomyopathy. Upon analyzing the genetic DNA of patients suffering from idiopathic dilated cardiomyopathy-related heart failure and rhythm abnormalities, two mutations in ABCC9 were detected. This gene encodes the cardiac KATP channel's regulatory SUR2A subunit. The intrinsic ATP hydrolytic cycle conformational rearrangement seen by mutant SUR2A proteins resulted in aberrant KATP channel phenotypes with impaired metabolic signal decoding. These channel abnormalities were described as vulnerability pathways to dilated cardiomyopathy.

## Conclusions

Insulinomas are neuroendocrine tumors primarily arising from the pancreas, specifically from the beta cells producing insulin. These tumors are typically benign, solitary, and relatively small, contributing to their generally favorable prognosis following surgical removal. When patients undergo surgery to excise insulinomas, they often experience a significant reduction or complete resolution of symptoms, leading to reasonable cure rates. One of the key aspects of insulinomas is their impact on the body's metabolic system, mainly of hyperinsulinemia. This condition occurs when excess insulin is released into the bloodstream, which can lead to recurrent episodes of hypoglycemia or low blood sugar levels. Patients may experience a range of symptoms during these hypoglycemic episodes, including weakness, confusion, sweating, palpitations, and, in severe cases, loss of consciousness. Interestingly, a growing body of evidence suggests a correlation between insulinomas and certain cardiac conditions. The secondary effects of sustained hyperinsulinemia and frequent hypoglycemic events may contribute to cardiovascular complications. For instance, repeated low blood sugar episodes can stress the heart and may alter cardiac function, potentially leading to heart failure over time due to increased myocardial oxygen demand. Insulinoma could be a reversible cause of heart failure, but more studies are needed to support this assumption further. Further research should clarify the mechanisms by which insulinoma affects cardiac health and explore whether early diagnosis and treatment of insulinoma could benefit heart function. Understanding the interplay between insulinoma and heart failure is essential for developing effective management strategies for patients affected by both conditions. Until more conclusive evidence is available, the notion that insulinoma could be a reversible cause of heart failure remains an area ripe for exploration and warrants rigorous investigation in future studies.
